# Congenital Central Hypothyroidism Caused by a Novel Thyroid-Stimulating Hormone-Beta Subunit Gene Mutation in Two Siblings

**DOI:** 10.4274/jcrpe.4595

**Published:** 2017-09-01

**Authors:** Bayram Özhan, Özlem Boz Anlaş, Bilge Sarıkepe, Burcu Albuz, Nur Semerci Gündüz

**Affiliations:** 1 Pamukkale University Faculty of Medicine, Department of Pediatrics, Division of Pediatric Endocrinology, Denizli, Turkey; 2 Pamukkale University Faculty of Medicine, Department of Medical Genetics, Denizli, Turkey

**Keywords:** hypothyroidism, Congenital, thyrotropin deficiency

## Abstract

Congenital central hypothyroidism (CCH) is a very rare disease. Alterations in pituitary development genes as well as mutations of immunoglobulin superfamily member 1 and transducin β-like protein 1 can result in CCH and multiple pituitary hormone deficiencies. However, mutations of the thyrotropin-releasing hormone receptor or thyroid-stimulating hormone-beta (TSHB) gene are responsible for isolated CCH. In this paper, we present the cases of two siblings with a novel mutation of TSHB. Direct sequencing of the coding regions and exon/intron boundaries of the TSHB gene revealed two homozygous nucleotides changes. One of them was c.40A>G (rs10776792) which is a very common variation that is also seen in healthy individuals, the other was c.94G>A at codon 32 of exon 2 which resulted in a change from glutamic acid to lysine (p.E32K). Both patients were homozygous and the parents were heterozygous.

**What is already known on this topic?**

Congenital central hypothyroidism (CCH) is a very rare disease. Genes included in pituitary development, as well as mutations of the immunoglobulin superfamily member 1 and transducin β-like protein 1 can cause syndromic CCH. However, isolated CCH is caused by mutations of the thyrotropin-releasing hormone receptor or thyroid-stimulating hormone-beta subunit genes.

**What this study adds?**

This article presents a novel mutation of TSHB genes in 2 patients with CCH.

## INTRODUCTION

Congenital central hypothyroidism (CCH) is a very rare disease associated with insufficient thyroid-stimulating hormone (TSH; also called thyrotropin) stimulation of a normally located thyroid gland. Alterations in pituitary development genes, such as PIT1, PROP1, HESX1, LHX3, LHX4, and SOX3 can result in CCH and multiple pituitary hormone deficiencies (1). Recently, mutations of the immunoglobulin super family member 1 (IGSF1) and transducin β-like protein 1 (TBL1X) genes have also been described as causes (2,3). IGSF1 is located on Xq 26.2 and its product may act as a signal transduction molecule in the hypophysis. Its precise physiological function is not known, but a deficiency of IGSF1 protein results in deficiencies of TSH and prolactin, pubertal delay and macroorchidism after adolescence (2). In cases with TBL1X gene mutations, hearing loss often accompanies central hypothyroidism (3).

Apart from these syndromic CCH causes, isolated CCH can be caused by mutations of the thyrotropin-releasing hormone (TRH) receptor, the least frequent one, or of TSH-beta (TSHB) subunit genes. The TSHB gene is located on chromosome 1p13 and contains 3 exons. The second and third exons encode a mature protein of 112 amino acids (4).

In this paper, we report two siblings with congenital secondary hypothyroidism who were diagnosed at the ages of 16 and 20 with a new mutation in the TSHB-subunit gene.

## CASE REPORTS

### Case 1

A 16-year-old girl was admitted to the hospital with a 20-day history of persistent vaginal bleeding. The patient was born to non-consanguineous Turkish parents following an uneventful, full-term second pregnancy. Her past medical history revealed that she was developmentally delayed and that she had received L-thyroxine (T_4_) treatment for a short period at the age of 2; however, her parents did not continue the treatment. She had 3 siblings. Two of them are dizygotic twins whose ages are 5.26 years. The sister’s height is 100.2 cm (Z-score; -1.22), weight 16.2 kg (Z-score; -1.22) and her brother’s height is 106.3 cm (Z-score; -1.22), weight 23 kg (Z-score; 1.26). Her oldest brother has short stature.

The physical examination revealed a myxedematous face, thin hair, dry skin, a distended abdomen with an umbilical hernia, and psychomotor retardation. Her weight was 26 kg [standard deviation (SD) score -7.46], and she had a height of 96 cm (SD score -10.45), a body mass index of 28.26 kg/m^2^, a blood pressure of 88/43 mmHg, and a pulse rate of 117 bpm. Her thyroid gland was not palpable. Her breast development was consistent with Tanner stage 3, she had no pubic or axillary hair.

The laboratory test results (Table 1A) showed that her creatinine phosphokinase, serum aspartate transaminase, alanine transaminase, triglyceride, and cholesterol levels were elevated. Her kidney function was within normal limits. The complete blood count revealed that she had severe anemia with a hemoglobin level of 5.6 g/dL associated with a deficiency of both iron and vitamin B12.

**Figure 1:**
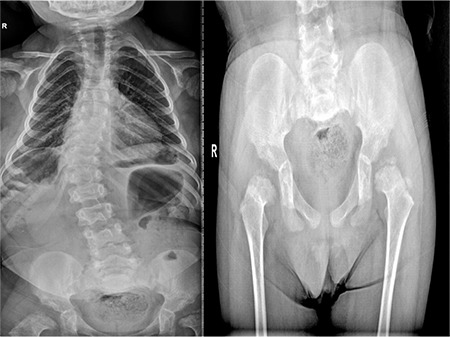
Epiphyseal dysgenesis and kyphoscoliosis in case 1

Her free T_4_ level was 0.05 ng/dL (0.8-2.2), and TSH level was 0.454 uIU/mL (0.51-4.30); prolactin (PRL) level was 15 ng/mL (4.79-23.3). Her follicle-stimulating hormone level (FSH) was 6.10 mIU/mL (0.5-2.41), luteinizing hormone (LH) level was 12.64 mIU/mL (0.18-0.3), and estradiol level 46.08 ng/mL (12.5-166). Insulin-like growth factor 1 level was 38.7 ng/mL (226-903). Radiological investigations revealed delayed bone age, epiphyseal dysgenesis, and kyphoscoliosis (Figure 1). Ultrasonography of her pelvis showed follicles in both ovaries, a uterine length of 57 mm, and an endometrial thickness of 7 mm.

Serum samples were obtained at 0, 20, 40, and 60 minutes after intravenous TRH (200 μg/m^2^) administration to evaluate her PRL and TSH responses (Table 1B). Her PRL response was normal, but there was no increase in TSH. Based on these physical findings and on the laboratory results which included low levels of free T_4_ and TSH, a diagnosis of secondary hypothyroidism was made. The pituitary gland was found to be normal in a hypothalamus-pituitary magnetic resonance imaging. To check the integrity of the hypothalamic-pituitary-adrenal axis, a low-dose adrenocorticotropic hormone stimulation test was performed; her peak cortisol level was 21.38 µg/dL. An echocardiogram was performed and it was normal. Usually, bradycardia accompanies hypothyroidism, but in our patient, tachycardia and hypotension were present due to severe anemia. L-T_4_ treatment was started. Her L-T_4_ dose was gradually increased from a quarter of the calculated dose to 100 µg/m^2^/day. Treatment with vitamin B12 and iron was also initiated for anemia.

### Case 2

**Figure 2:**
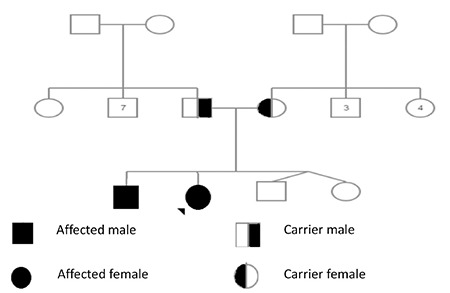
Pedigree of the family

The 20-year-old brother of Case 1 was also affected (Figure 2). He had coarse facial features, dry skin, cold extremities, and moderate intellectual disability. His weight was 46.4 kg (SD score -3), and he had a height of 133.4 cm (SD score -6), a blood pressure of 100/80 mmHg, and a pulse rate of 70 bpm. His thyroid gland was not palpable. Testicular volume was 20 mL bilaterally and his pubic hair was consistent with Tanner stage 4. His baseline free T_4_ level was 0.114 ng/dL (0.8-2.2), TSH level was 1.93 IU/mL (0.51-4.30), and PRL level 30 ng/mL (4.79-23.3). His FSH level was 2.35 mIU/mL (1.5-12.4), LH level 3.98 mIU/mL (1.7-8.6), and total testosterone level was 5.81 ng/mL (2.8-8). His hemoglobin level was normal, and his cortisol level was 10.76 µg/dL. Insulin-like growth factor 1 level was 216 ng/mL (116-358). His bone age was consistent with age 15 years. Based on these physical findings and on the laboratory results (Table 1A), a diagnosis of isolated secondary hypothyroidism was made, and L-T4 therapy was initiated with a low dose and titrated with free T_4_ values to maintain the serum free T_4_ concentration in the upper 50 percent of the normal range.

In both patients, euthyroid status was achieved after the first month of therapy. Lipid profile and liver function test abnormalities of the first proband normalized and the anemia improved.

### Genetic Analyses

Genomic DNA was extracted from peripheral blood leucocytes of the patients and their family members using a commercially available DNA extraction kit (QuickGene DNA whole blood kit S, Japan). DNA quality control was checked using a spectrophotometer (Thermo Scientific NanoDrop 2000, USA). Specific primers were designed to amplify all the coding regions and the exon/intron boundaries of the TSHB gene using a polimerase chain reaction (PCR). For exon II, the forward primer was 5’-GGGATGGTACTGAAGTTTGGT-3’, and the reverse primer was 5’-AGATTTGGGAAATGAGGTTGTG- 3’; for exon III the forward primer was 5’-GGCTAAGCAATTCTTTCCCAGT-3’ and the reverse primer was 5’-GCTCTCTAACGCCTGTGTAGG-3’. ExPrime Taq Premix was used as the PCR master mix. The quality of the PCR reaction was analyzed using 2% agarose gel electrophoresis. An OMEGA bio-tek E.Z.N.A. Cycle Pure kit was used to purify the PCR products. The purified specific PCR products were then directly sequenced using a commercial kit according to the manufacturer’s instructions (GenomeLab DTCS-Quick Start Kit, USA; BECKMAN Coultur CEQ 8000 Genetic Analysis System, USA).

**Figure 3:**
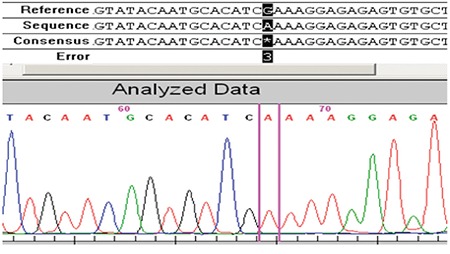
Homozygous c.94G>A

**Figure 4:**
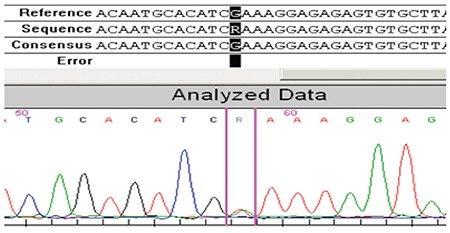
Heterozygous c.94G>A

Direct sequencing of the coding regions and the exon/intron boundaries for the TSHB gene revealed 2 homozygous nucleotide changes. The first C.40A>G (rs10776792) is a very common variation that can also be seen in healthy individuals, including the healthy members of the family of the two patients (the parents and twin siblings). The other nucleotide change was c.94G>A at codon 32 of exon 2, which results in a change of glutamic acid to lysine (p.E32K). For this novel mutation, both patients were homozygous and the parents were heterozygous (Figures 2, 3, 4). The PolyPhen 2 (score: 0.987), SIFT (score: 0), and Mutation Taster prediction tools (disease causing) all predicted that the mutation was pathogenic.

## DISCUSSION

We herein report two siblings with a new mutation of the TSHB gene in a non-consanguineous Turkish family.

The first mutation in TSHB was a single-base substitution in the 29^th^ codon resulting in the replacement of glycine by arginine (G29R) (5). Since the first description of TSHB gene mutation, additional mutations have been reported including missense (C108Y, C105R, and G49R), non-sense, frameshift (p.E32*, p.Q69*, p.C125Vfs*10, and p.F77Sfs*6), and splice-site (c.162G>A, c.1625G>A) mutations (1,5,6,7,8,9,10,11,12). The most commonly reported mutation is the C105Vfs114X mutation. This mutation is located on exon 3 of the TSHB gene, and it was first described in 1996 (10).

The first reported Turkish cases were two siblings who had a C-to-T transition at nucleotide 654 which led to the conversion of a glutamine into a premature stop codon in codon 49 (Q49X) (12). In 2004, the IVS2+5 G>A mutation resulting in isolated TSH deficiency was reported in 4 children from two consanguineous Turkish families (13). More recently, a homozygous TSHB deletion was found in a 51-day-old male of Turkish descent who was diagnosed during a prolonged jaundice investigation (14).

The mutation in our patients was c.94G>A at codon 32 of exon 2; it resulted in a change from glutamic acid to lysine (p.E32K). Another homozygous mutation at the same position (c.94G>T, p.E32*) was previously reported in 3 Greek children from two families (9). In silico analysis of c.94G>A revealed that this alteration was pathogenic. To the best of our knowledge, the mutation detected in our patients has never been reported in central corneal thickness. Unfortunately, we could not perform functional analyses, but the in silico analyses were in agreement that the variant has a disease-causing effect.

TSH, LH, and FSH are pituitary glycoprotein hormones. All three are composed of an alpha-subunit and a beta-subunit coupled with non-covalent bonds. Their alpha-subunits are identical, but their beta-subunits are hormone-specific and confer biological specificity. TSHB gene mutations cause alterations in the size or shape of the TSHB-subunit by affecting the beta-subunit’s seatbelt region or by changing the protein building blocks that are used to make the beta-subunit (1). The production or release of functional TSH from the pituitary gland is thus decreased. This TSH deficiency results in low hormone levels and leads to hypothyroidism. Consequently, TRH levels increase to stimulate both the production of TSH and PRL. There is no increase in TSH levels, because both alpha- and beta-subunits are needed to form intact TSH. On the other hand, serum alpha-subunit concentrations increase, a biochemical hallmark in TSHB gene mutations, and the PRL levels increase. For diagnostic purposes, in these patients, high serum alpha-subunit concentrations or a TRH stimulation test can be used to show isolated TSH deficiency and to indicate that the TRH receptor is intact based on an elevated PRL level (1). We performed a TRH stimulation test only for the index case, and the results were compatible with those for an isolated TSH deficiency. The elevated basal PRL levels and the very low TSH levels despite the severe hypothyroidism in the second case were also considered findings indicative of isolated TSH deficiency.

The clinical consequences of CCH are related to the severity and duration of thyroid hormone deprivation. The mutations probably have little impact on phenotype, and no genotype-phenotype correlation has been reported. All of the reported cases showed clinical signs of hypothyroidism, such as prolonged jaundice, coarse facies, large fontanelles, dry skin, umbilical hernia, enlarged tongue, mental and motor retardation. Both patients were in their late teens and both showed all of the clinical and metabolic signs of severe hypothyroidism. They also both had severe motor and mental retardation. The index case had kyphoscoliosis in addition to a very short stature. The radiological appearance was compatible with epiphyseal dysgenesis which is a hallmark of long-standing untreated hypothyroidism (15). Hypothyroidism is treated with L-thyroxine, but measurement of serum TSH cannot be used as a guide to the adequacy of T_4_ replacement therapy in CCH. Serum TSH is always suppressed to <0.1 mU/L, so dosage should be titrated with free T_4_ value to maintain the serum free T_4_ concentration in the upper 50 percent of the normal range (16).

Congenital hypothyroidism is the most common preventable cause of mental retardation. Screening programs for newborns were developed to detect this preventable condition as early as possible. Screening by T_4_ and TSH is highly sensitive. However, TSH-based screening is more frequently used around the world even though it often leads to central hypothyroidism being overlooked (17). Identification of a TSHB mutation would help in the genetic counseling and early diagnosis of siblings in affected families.

## Figures and Tables

**Table 1A:**
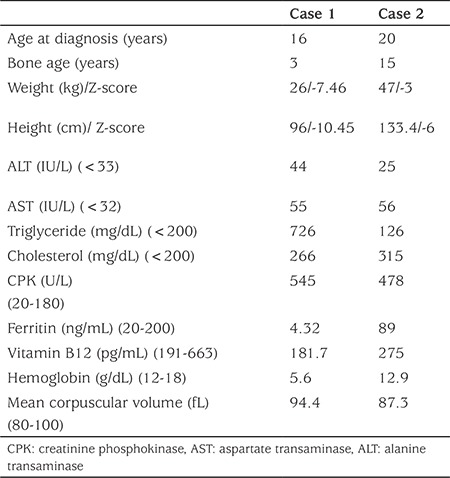
Anthropometric measurements and laboratory investigations in the two patients

**Table 1B:**
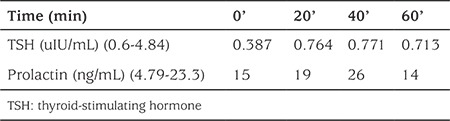
Thyrotropin-releasing hormone stimulation test results of the index case
